# Supplementary material for: Approaching future rewards or waiting for them to arrive: Spatial representations of time and intertemporal choice
Source: PLoS One. 2024 Apr 5;19(4):e0301781. doi: 10.1371/journal.pone.0301781 (PMC10997117; doi:10.1371/journal.pone.0301781)
Supplement: S1 Appendix — (DOCX) [file pone.0301781.s001.docx]

**S1 Appendix**

Table A. Pairwise comparisons for effect of spatial prime condition on discount factor

| Spatial prime comparison | Mean difference | SE | *p* |
| --- | --- | --- | --- |
| Control vs. Ego-moving | .059 | .026 | .023 |
| Control vs. Time-moving | .021 | .026 | .417 |
| Ego-moving vs. Time-moving | -.038 | .026 | .137 |

*Note.* SE = standard error of the mean difference. *p-*values not corrected for multiple comparisons.

Table B. Participant employment status

| Employment Status | Frequency | Percent |
| --- | --- | --- |
| Due to start a new job within the next month | 13 | 3% |
| Full-Time | 199 | 45% |
| Not in paid work | 48 | 11% |
| Other | 36 | 8% |
| Part-Time | 82 | 19% |
| Unemployed | 63 | 14% |

*Note.* Employment status data was unavailable from Prolific for 147 participants. Percentages are based on participants for which employment status data were available.

Table C. Binary logistic regression on Monday (time-moving) responses with age as a predictor

| Variables | Log odds | SE | Wald | *p* | OR |
| --- | --- | --- | --- | --- | --- |
| Age | .025 | .009 | 8.11 | .004 | 1.025 |
| Constant | .376 | .294 | 1.63 | .202 | 1.456 |

*Note.* SE = standard error of log odds. OR = odds ratio.

Table D. Independent samples t-tests: Mean discount factor, perceived wait time, and perceived control by Monday-Friday responses

| Variable | Monday | | Friday | | *p* |  |  |
| --- | --- | --- | --- | --- | --- | --- | --- |
|  | *M* | *SE* | *M* | *SE* |  | *t* | *df* |
| Discount factor | 0.42 | 0.01 | 0.39 | 0.02 | .20 | -1.31 | 535 |
| Perceived wait time | 6.35 | 0.09 | 6.37 | 0.16 | .89 | 0.14 | 539 |
| Perceived control | 4.06 | 0.12 | 4.14 | 0.22 | .73 | 0.35 | 541 |

*Note.* SE = standard error of the mean
